# Defining the Participatory Aspect in Health and Dementia Care Research

**DOI:** 10.1093/geroni/igaa057.2857

**Published:** 2020-12-16

**Authors:** Martina Roes

**Affiliations:** German Center for Neurodegenerative Diseases (DZNE), Witten, Germany

## Abstract

In the last decade, the terms ’engagement’, ‘involvement’, ‘patient-public involvement (PPI)’ and/or ‘participatory research’ have been increasingly used in literature. However, it seems that each author has their own definition of the terms and there is not consensus on what constitutes engagement, involvement or participation in health care research. In a broad sense, it seems that PPI can be understood as a co-design strategy, but the terms are not used consistently. Definitions of participation in research tend toward describing the methodological aspect of research rather than mapping appropriate use of methods. Representation of the diversity of society is also challenging and involvement of people with dementia is often neglected. To improve participatory research and outcomes, it is valuable to understand applied methods. Therefore, we conducted a meta-analysis of literature reviews that analyzed methodological concepts and application of these participatory concepts. This presentation will provide key results of this review. Part of a symposium sponsored by the Patient/Person Engagement in Research Interest Group.

